# *MEX3C* induces cognitive impairment in mice through autophagy inhibition

**DOI:** 10.1002/brb3.3245

**Published:** 2023-08-31

**Authors:** Kai Wang, Hao‐Nan Zhang, Yong Du

**Affiliations:** ^1^ School of Clinical Medicine Ningxia Medical University Ningxia Yinchuan China; ^2^ Department of Pediatric Surgery General Hospital of Ningxia Medical University Ningxia Yinchuan China

**Keywords:** autophagy, cognitive impairment, MEX3C protein

## Abstract

**Background:**

The muscle excess 3 (*MEX3C*) protein comprises one of two conserved KH hnRNP K homology domains of the *Caenorhabditis elegans* protein family, a gene involved in the metabolism of key RNAs at posttranscriptional levels during the development of *C. elegans*, but its function in mammals is unclear.

**Methods and results:**

In this study, we found that *MEX3C* plays a key role in learning and cognitive function. The learning and cognitive abilities of *MEX3C*‐knockout (KO) mice were significantly decreased relative to those of wild‐type (WT) mice in behavioral experiments, including the shuttle box, Morris water maze, and new object recognition. Nissl staining showed a decrease in the number of Nissl bodies and in the maturation of hippocampal and cortical neurons. A Western blot analysis of the neuron‐specific nuclear (NeuN) protein NEUN protein showed that the expression of that protein was decreased, which was consistent with the results of Nissl staining. Of note, the expression of sequestosome I p62 and Parkin BCL‐2‐associated X (Bax) Bax and B‐cell lymphoma‐2 (Bcl‐2) Bcl‐2 proteins also showed a downward trend, suggesting that the *MEX3C* gene may cause a decrease in the number and maturity of neuronal cells by increasing apoptosis through the inhibition of autophagy. In addition, Golgi staining showed that the complexity of neurons in the hippocampus and cerebral cortex was reduced, and the postsynaptic density protein 95 and growth‐associated protein (GAP‐43) also showed different degrees of reduction.

**Conclusion:**

The KO of the *MEX3C* gene reduces the plasticity of synapses in various regions of the hippocampus, thereby affecting the function of the hippocampus and eventually causing the decline of cognitive function. On the other hand, compared with WT mice, *MEX3C*‐KO mice showed increased anxiety‐like behaviors in minefield and elevated plus maze tests.

## Graphical abstract

Mex3c KO leads to cognitive decline and anxiety in mice due to reduced neuron number and synapse plasticity.

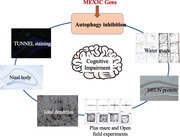

## 1 INTRODUCTION

The continuous development and improvement of the brain is the most important part of the human evolution process, and it is also the key to distinguishing humans from other mammals. The human brain exhibits unique and complex characteristic functions, such as language, tool use, self‐emotion, and well‐developed cognitive learning functions. The expression and function of different genes underpin the molecular basis of the human brain (Langston et al., 2010; Olson & Varki, 2003). Nervous system development includes two main stages. The first is the prenatal stage, which is determined by the maternal environment and genetic information. The second is the postnatal stage when the proliferation and differentiation of the nervous system are in a period of extreme sensitivity to external environmental stimuli, extreme plasticity, and long‐term development (Kolb et al., 2014).

A Nissl body is a unique neuronal structure. Its main function is to synthesize related proteins, such as the renewal of certain components in neuron cells and the production of neurotransmitters and enzymes, which is one of the indicators reflecting the degree of neuronal activity. Under physiological conditions, autophagy degrades abnormal substances and participates in maintaining neuronal homeostasis. Recent studies have revealed that autophagy plays a critical role in immunity, balancing beneficial, and detrimental effects. Autophagy dysfunction is associated with multiple inflammation‐related diseases (Levine et al., 2011). In the central nervous system, autophagy defects cause neurodegeneration in mice. Previous studies have shown that autophagy defects in neurons are linked with the development of neurodegeneration (He & Klionsky, 2006). Autophagy can also improve cognitive function by maintaining the blood–brain barrier. In addition, presynaptic‐ and postsynaptic‐related proteins are key targets in synaptic plasticity research.

Growth‐associated protein (GAP)‐43 is a neuron‐specific phosphoprotein that is widely distributed in the cerebellum, spinal cord, and neuron cells, and its main function is to promote the growth and development of neurons and the regeneration of axons (Doster et al., 1991). Therefore, GAP‐43 has been considered the preferred probe for exploring neural plasticity, including neuronal growth, development, and injury repair, and as a molecular marker for neuronal growth, axonal regeneration, and synaptic reconstruction.

Postsynaptic density protein 95 (PSD‐95) is an abundant scaffold protein that was first identified in the dense region of the postsynaptic membrane and is a member of the membrane‐associated guanylate kinase protein superfamily (Tavares et al., 2001), which affects synaptic structural plasticity and functional plasticity. Therefore, the upregulation of GAP‐43 and PSD‐95 expression can enhance synaptic connection, improve neural function, protect neurons, and thus promote tissue repair and brain remodeling.

Muscle excess 3 (*MEX3C*) is the mammalian homologue of the RNA‐binding protein of *Caenorhabditis elegans* (Jasinski‐Bergner et al., 2020). The N terminus of *MEX3C* contains two tandem KH domains and a RING domain with ubiquitin ligase activity at the C terminus. *MEX3C* has been shown to be associated with a variety of diseases, and *MEX3C* homozygous mutant mice exhibit growth retardation and perinatal lethality due to IGF‐1 deficiency in bone (Jiao et al., 2012). Circulating and bone‐localized IGF‐1 protein levels were significantly reduced in *MEX3C* homozygous mutant mice, but IGF‐1 mRNA levels were not (Jiao et al., 2012). When we observed the postnatal growth of *MEX3C* knockout (KO) mice, we found that homozygous KO mice not only had growth retardation but also exhibited a difference in behavior (evading the experimenter's grasp) compared with control mice of the same age.

Studies have revealed that *MEX3C* plays an important role in the development of the embryonic neural tube (Du et al., 2020). It may participate in the regulation of the proliferation and apoptosis of NSCs through the interaction of neuroactive ligand receptors, TGF‐β, Notch, and other signaling pathways before affecting the neural tube.

However, there is a lack of research on autophagy‐related processes. It is widely believed that *MEX3C* is highly expressed in the brain, and the hippocampus in the brain is the main area responsible for cognitive functions, such as learning and memory (Carroll, 2003). Motivated by the differential behavior of *MEX3C* mutant mice and the higher expression levels in the brain, we investigated the possible role of the *MEX3C* gene in the brain and hippocampus and revealed the possible functions of the autophagy process therein.

## 2 MATERIALS AND METHODS

### 2.1 Animals

We selected 7‐week‐old (22 ± 2 g) mice for the experiments. Wild‐type (WT) C57BL/6J mice were obtained from the Ningxia Medical University Laboratory Animal Centre. *MEX3C*‐KO mice with a C57BL/6J background were purchased from Saiye Experimental Animal Technology Co., Ltd. (SCXK 2018‐0032). The *MEX3C*‐KO mice were genotyped as RESULT 2 described, and all animals were housed in a specific pathogen‐free environment. 20 *MEX3C*‐KO and 20 WT mice were used in the experiments. A light‐dark cycle was implemented at intervals of 12 h, the temperature was controlled at 25°C, and all mice had free access to water. All experimental protocols were approved by the Medical Research Ethics Review Committee of Ningxia Medical University General Hospital (ethics no. KYU‐2022‐0028).

### 2.2 Behavioral assessment

#### 2.2.1 New object recognition test

The experimental site was an open field with dimensions of 50 (length) × 50 (width) × 30 cm (height). We placed two identical yellow spheres (objects 1 and 2) on one side of the field, 25 cm apart, 12.5 cm from the side wall, with a 5 cm radius from the center of the object to provide exploration and capture areas (33505354). The whole experiment was performed in three stages: In the first stage, 24 h before the start of the experiment, each mouse freely explored an open field without objects for 5 min. In the second stage, objects 1 and 2 were positioned, and the mice explored freely for 6 min. Then, the mice were removed. In the third stage, after 60 min, one of the yellow spheres (object 1) was replaced with a different shape (red cone) (object 3), and the mouse was placed in the same position as in the second stage to explore freely for 6 min. The exploration time was defined as the time when the mouse's nose touched or pointed to an object in the exploration capture area. The total time an animal spent exploring familiar and novel objects was recorded and compared using a recognition index (RI): RI = (Tobject3)/(Tobject3 + Tobject2), where *T* = exploration time (28463798). The higher the RI value, the higher the memory retention rate. The venue and objects were cleaned with 70% ethanol after each mouse task to prevent the accumulation of olfactory cues.

#### 2.2.2 Morris water maze test

The water maze experimental device was a circular pool (diameter: 80 cm and height: 50 cm) filled with tap water at 23 ± 2°C and nontoxic white dye and was equipped with a round black platform (diameter: 5 cm) for escape. The circular pool was divided into four equal quadrants. The circular platform was placed in the first quadrant (the target quadrant), and the remaining quadrants were marked clockwise as the second, third, and fourth quadrants. The experiment lasted 7 days in total, and the time interval between each experiment was 24 h. A complete test consisted of three parts: a platform‐visible period, a platform‐hidden period, and a no‐platform period. On the first day, the platform was visible, the water surface was adjusted to be 1 cm lower than the platform, and the mice were allowed to explore freely in the water for 60 s. In the hidden‐platform test (days 2–6), the platform was submerged 1.0 cm below the water surface. A mouse was placed on the outside of each quadrant and was allowed to swim freely, with the experiment ending when the mouse found the hidden platform (5 s on the platform) or when the time (60 s) ran out. If the platform was not found after 60 s, the mouse was guided to find the platform and was allowed to remain there for 10 s. The seventh day was the platform‐free period. The original platform was removed, and the mice were allowed to enter the water in the third quadrant. The trajectory, the times of crossing the original platform, and the incubation period were recorded and compared.

#### 2.2.3 Shuttle box active avoidance test

The experimental setup consisted of two transparent compartments with a door in the middle (MED Associates Inc.). At the beginning of the experiment, the mice were placed in a side chamber to adapt to the chamber environment for 10 s; then, they were given conditioned stimulation (photoacoustic stimulation for 5 s). After 5 s, if the mouse did not pass through the gate, an unconditioned stimulus (electric shock, 0.3 mA, 50 Hz, 10 s) was given. If the mouse still did not pass through the gate, the experiment was stopped after a resting period of 10 s, and the next experiment was started. Active avoidance was determined when a mouse crossed the gate after the conditioned stimulus and before the unconditioned stimulus. The time it took from the start of the experiment to when the mouse passed through the gate after receiving the conditioned stimulus was termed active avoidance latency. On the fifth day, the mouse memory test was carried out, and the active avoidance latency time and the number of times of active avoidance were detected and recorded. In this experiment, we used MED‐PC Version V (MED Associates Inc.) to record the above experimental data.

#### 2.2.4 Elevated plus maze

The experimental setup of the elevated plus maze consisted of two open arms and two closed arms perpendicular to each other (elevation 50 cm from the ground; wall height 15 cm). Each mouse was placed in the central area of the elevated plus maze (10 × 10 cm) facing the open arms and was allowed to freely explore the maze for 5 min (29157601); the mice were recorded using a digital video camera and analyzed using SAMRT 3.0 behavioral analysis software. The time each mouse spent in the open arm and the closed arm and the number of entries were recorded. After each trial, the equipment was cleaned with 70% ethanol. Anxiety‐like behavior was assessed by the ratio of open‐arm dwell time to total time (open‐arm dwell time + closed‐arm dwell time).

#### 2.2.5 Open‐field test

Briefly, each animal was placed in the central area of an open blank white field (50 [length] × 50 [width] × 30 cm [height]) and was allowed to explore freely for 5 min. The trajectory of the mice during exercise was recorded. After each experiment, the site was cleaned with 75% ethanol. Anxiety‐like behaviors were assessed by comparing dwell times in the central zone (central length × width 25 cm).

### 2.3 Tissue processing

After the behavioral experiment, the mice were anesthetized with isoflurane, fixed with 4% paraformaldehyde through the heart, and the whole brain was immediately removed and fixed in new 4% paraformaldehyde at 4°C for 12 h. Then, the brain tissue was transferred to 70% alcohol for dehydration for 24 h, followed by standard procedures for dehydration and paraffin embedding. The prepared wax blocks were stored in a −20°C refrigerator for long‐term storage. Sections of 3 μm were cut using a paraffin microtome and placed on glass slides.

### 2.4 Western blot analysis

Proteins from the hippocampus and cerebral cortex were extracted using a whole‐protein extraction kit (Jiangsu KGI Biotechnology Co., Ltd.), and the protein concentration was detected using a BCA protein concentration assay kit (Jiangsu KGI Biotechnology Co., Ltd.). Equal amounts of protein (40 μg per reagent tube) were separated with 10% SDS–PAGE and transferred to a 0.45 μm PVDF membrane. Then, the samples were blocked with 5% BSA at room temperature (RT) (25°C) RT for 1 h. NeuN (ab177487, Abcam; dilution, 1:1000); PSD95 (ab18258, Abcam; dilution, 1:1000); GAP‐43 (ab75810, Abcam; dilution, 1:1000); p62 (GB11531, Servicebio; dilution, 1:1000); Parkin (GB114834, Servicebio; dilution, 1:1000); Bax (ab32503, Abcam; dilution, 1:1000); Bcl‐2 (ab182858, Abcam; dilution, 1:1000) were incubated with the primary antibody at 4°C for 16 h; then, the membrane was washed with Tris‐buffered saline (TBS) containing 5% Tween 20, and the secondary antibody Alexa Fluor 790 (ab186695, Abcam; dilution 1:10,000) and IRDye 680RD (ab216777, Abcam; dilution 1:10,000) were incubated for 1 h at RT (25°C). All antibodies were diluted in TBS buffer containing 5% Tween 20. The protein signal was quantitatively analyzed using ImageJ 9.0 software. All experiments were performed at least three times.

### 2.5 Nissl staining (Lindroos & Leinonen, 1983)

First, the paraffin sections were subjected to conventional dewaxing in xylene for 5–10 min; then, they were dewaxed in fresh xylene for another 5–10 min. Next, they were treated with ethanol for 5 min and washed with water for 2 min. Then, Nissl staining solution (Wuhan Xavier Biotechnology Co., Ltd.) was used to stain the sections for 3 min. Finally, the coverslips were scanned and photographed, and ImageJ 9.0 software was used for quantitative analysis.

### 2.6 Evaluation of apoptosis with terminal deoxynucleotidyl transferase‐mediated dUTP nick‐end labeling (TUNEL) staining

To detect cell apoptosis, we chose to use a one‐step TUNEL cell apoptosis detection kit (China Jiangsu KGI Biotechnology Co., Ltd., catalogue no. KGA7071). The experimental procedure was to dewax the paraffin sections of the tissue samples according to the conventional method, add 100 μL of proteinase K working solution dropwise to each tissue section, react at 37°C for 30 min, add 100 μL of DNase I reaction solution dropwise, and treat at 37°C for 30 min. Then, 50 μL of TdT enzyme reaction solution was added dropwise to the sample, which was placed in a warm box and reacted in the dark at 37°C for 60 min. Finally, 50 μL of streptavidin–fluorescein labeling solution was added dropwise, and the sample was placed in a wet box and reacted at 37°C in the dark for 30 min; next, it was counterstained with DAPI staining solution to show the cell nuclei. Finally, a high‐resolution digital slide scanning imaging analysis system (Leica Application Suite X & Leica DM6 M LIBS) was used to capture images, which were then analyzed.

### 2.7 Statistical analysis

All values were presented as the mean ± standard deviation (SD). Statistical analyses were performed using *t* tests for two groups, and an analysis of variance was used for the shuttle box experiment. A value of *p* ≤ .05 was considered significant. Error bars represent SD.

## 3 RESULTS

### 3.1 Expression and localization of the *MEX3C* gene in the mouse brain and hippocampus

**FIGURE 1:**
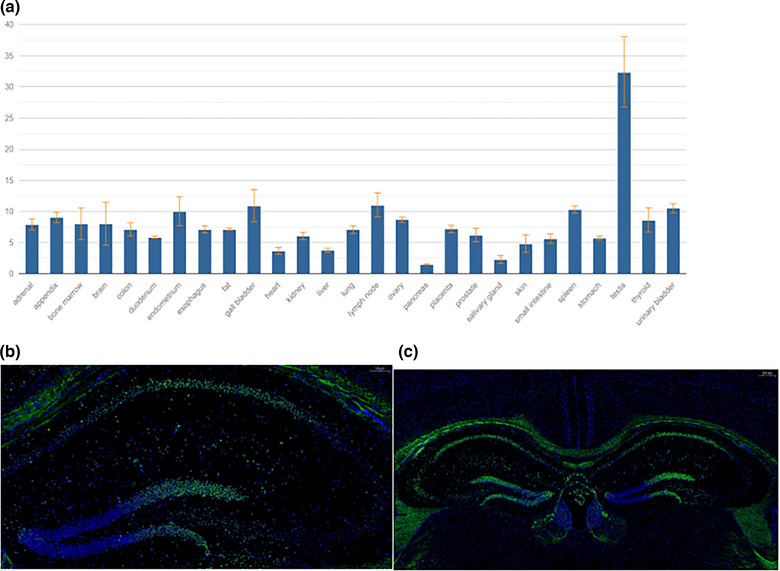
(a) The expression level of *MEX3C* gene in various tissues and organs. (b and c) By in situ hybridization analysis, the *MEX3C* gene is mainly highly expressed in the hypothalamus, cortex, and hippocampus of mice.

To explore the potential role of the *MEX3C* gene in the mouse brain and hippocampus, we first analyzed the expression levels of the *MEX3C* gene in the mouse brain and other tissues and organs by data mining the NCBI database. The analysis results showed that the expression level of the *MEX3C* gene in the mouse brain was much higher than that in other tissues (Figure 1a). To clarify the specific expression pattern of the *MEX3C* gene in the mouse brain, we explored the expression of the *MEX3C* gene in various functional areas of the mouse brain by in situ hybridization. The *MEX3C* gene is mainly expressed in the mouse hypothalamus, cerebral cortex, and hippocampus (Figure 1b,c). This suggests that the *MEX3C* gene may play an important role in maintaining the normal learning and cognitive functions of the cerebral cortex and hippocampus.

### 3.2 Genotype identification of *MEX3C*‐gene‐knockout mice

**FIGURE 2:**
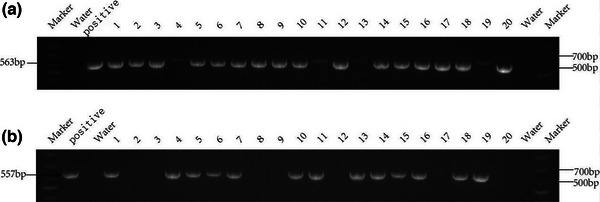
PCR genotyping results (a) primer R1 (b) primer R2.

To further analyze whether the loss of the *MEX3C* gene causes damage to the learning, cognitive function, and behavior of the mouse brain, we first knocked out the mouse *MEX3C* gene by using CRISPR‐Cas9 gene‐editing technology to obtain homozygous *MEX3C*‐KO mice. The genotype of offspring mice was identified by PCR technology. For the PCR, we designed two primers (R1 and R2), and the genotype of the mice was determined according to the results of two PCRs (Figure 2a,b). If only R1 (563 bp) was positive, it was deemed a homozygous KO mouse (*Mex3c* ^−/−^); if only R2 (557 bp) was positive, it was deemed a WT mouse (*Mex3c* ^+/+^); if both R1 (563 bp) and R2 (557 bp) were positive, it was deemed a heterozygous mouse (*Mex3c* ^+/−^). Finally, we selected 28 *Mex3c* ^−/−^ (KO group) and *Mex3c* ^+/+^ (WT group) mice according to the random number table method for the subsequent related experiments.

### 3.3 Genetic deletion of the *MEX3C* gene can cause cognitive and memory impairment in mice

**FIGURE 3:**
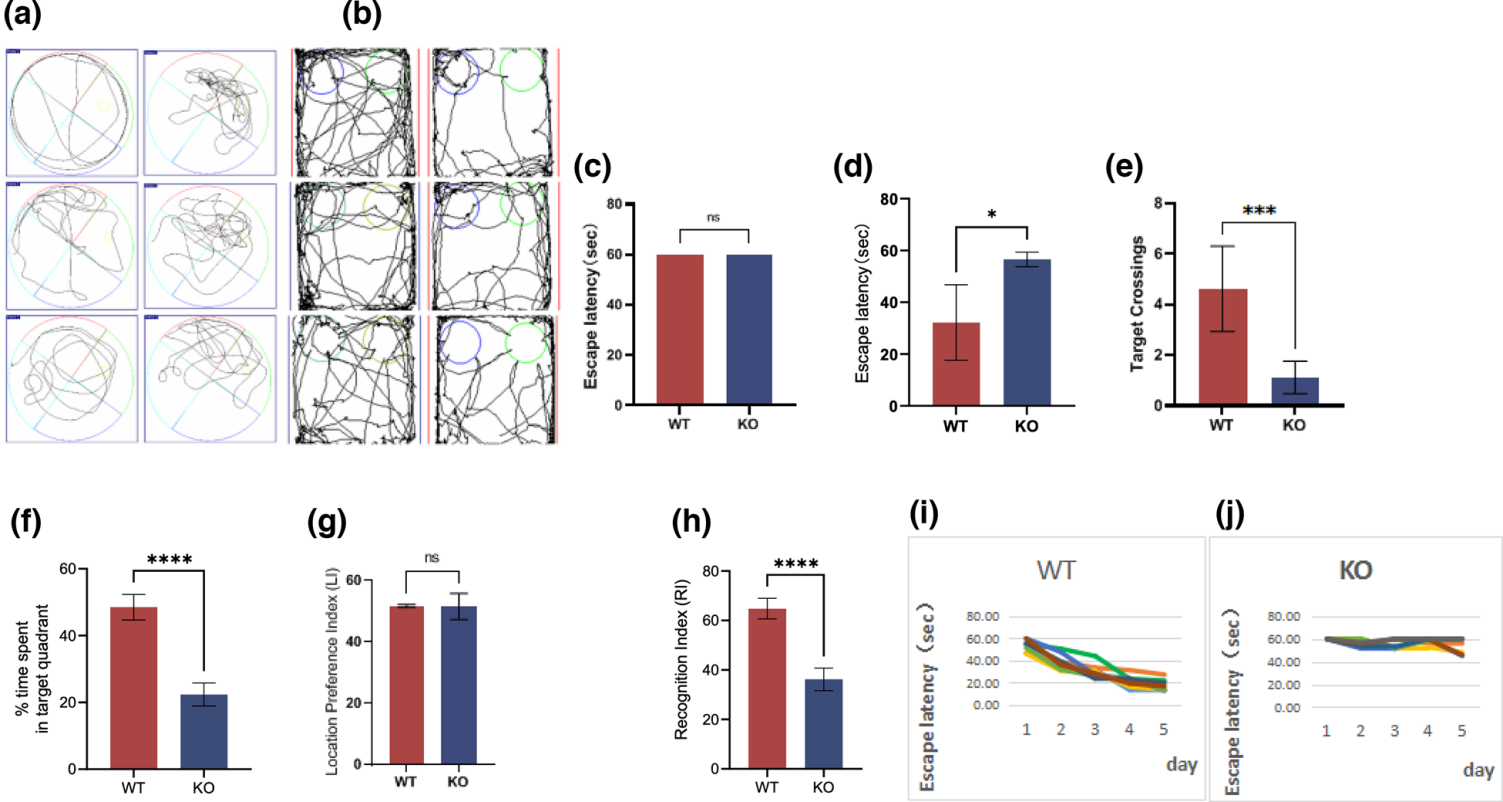
The results of mouse water maze and novel object recognition experiments show that *MEX3C* knockout (KO) mice have cognitive decline. (a) Water maze activity trajectory: the left side is the no‐platform trajectory of the mice in the KO group, and the right side is the no‐platform trajectory of the wild‐type (WT) group mice. After 6 days of training, the mice in the KO group had no obvious tendency to explore the target quadrant or the area where the platform was located, indicating that the mice in the KO group did not produce relevant memory or had weak memory recall ability; (b) Activity track of new object recognition experiment; the left side is the track map of WT mice, and the right side is the track map of KO mice. The mice in KO group have no difference in exploring new and old objects, that is, there is no memory or weak memory for old objects; (c) The escape latency of the visible stage of the water maze platform (WT, *n* = 20, *t* = 60 ± 0 s; KO, *n* = 20, *t* = 60 ± 0 s); (d) The 5‐day average escape latency of the hidden‐platform stage (WT, *n* = 8, escape time = 32.18 ± 14.57 s; KO, *n* = 8, escape time = 47.46 ± 2.82 s); (e) Times of crossing the platform area during the no‐plateau period (WT, *n* = 8, times = 4.63 ± 1.69; KO, *n* = 8, times = 1.13 ± 0.64, *p* = .0002); (f) Duration of the platform area during the no‐platform period percentage (WT, *n* = 8, time percentage = 48.52% ± 3.83%; KO, *n* = 8, time percentage = 22.39% ± 3.46%, *p* < .0001); (g) LI value of new object recognition experiment (WT, *n* = 8, LI = 51.72% ± 2.24%; KO, *n* = 8, LI = 51.51 ± 4.24, *p* = .8413); (H) recognition index (RI) value of new object recognition experiment (WT, *n* = 8, RI = 64.8% ± 4.15%; KO, *n* = 8, RI = 36.21% ± 4.56%, *p* < .0001); (i) Escape time of mice in WT group during hidden‐platform period; (j) Escape time of mice in KO group during hidden‐platform period. The data are mean ± standard deviation (SD). ^ns^ *p* > .05, *p < .05, ***p < .001, ****p < .0001.

As the expression of the *MEX3C* gene in the mouse central nervous system is much higher than in other tissues, to clarify the regulation of this gene on the brain, a series of behavioral experiments were designed using previously bred *MEX3C* ^+/+^ (WT) and *MEX3C* ^−/−^ (KO) mice. First, we evaluated their cognitive, learning, and memory abilities, and the KO group showed significant deficits in spatial and recognition memory. In the Morris water maze test, the first day of adaptive training (the platform‐visible period) revealed no significant difference between the two groups of experimental mice (Figure 3a), and there was no abnormality in their visual acuity and motor ability (Figure 3b). This was followed by a 5‐day positioning training. In the experiment (platform‐hidden period), it was found that compared with the WT mice, the escape latency of the KO mice did not reduce with increased training times, and the average value of the 5‐day escape latency was significantly longer than that of the WT mice (average latency: WT mice: 32.18 ± 3.90; KO mice: 55.91 ± 2.38 s) (Figure 3c). In the space exploration experiments (no‐platform phase), the KO mice exhibited a shorter dwell time in the target quadrant and fewer escape platform crossings than the WT mice (Figure 3d).

Given the experimental results in the water maze, we questioned whether the model would achieve the same results in the novel object recognition experiment and the shuttle box experiment (Figure 3e,f). The results of the novel object recognition experiment showed no significant difference in the exploration preferences of the WT mice and the KO mice for objects in different locations (Figure 3g), which indicated that both groups of mice had similar curiosity and motivation to explore objects. However, in the subsequent memory test, compared with the WT mice, the exploration time and new object RI of the KO mice were significantly decreased (Figure 3h), and the memory ability of the KO mice for old objects was also decreased (Figure 3i,j). Combining the above two experimental results, we determined that the deletion of the *MEX3C* gene leads to impairment of the learning and memory ability of mice.

### 3.4 Genetic deletion of the *MEX3C* gene causes increased anxiety in mice

**FIGURE 4:**
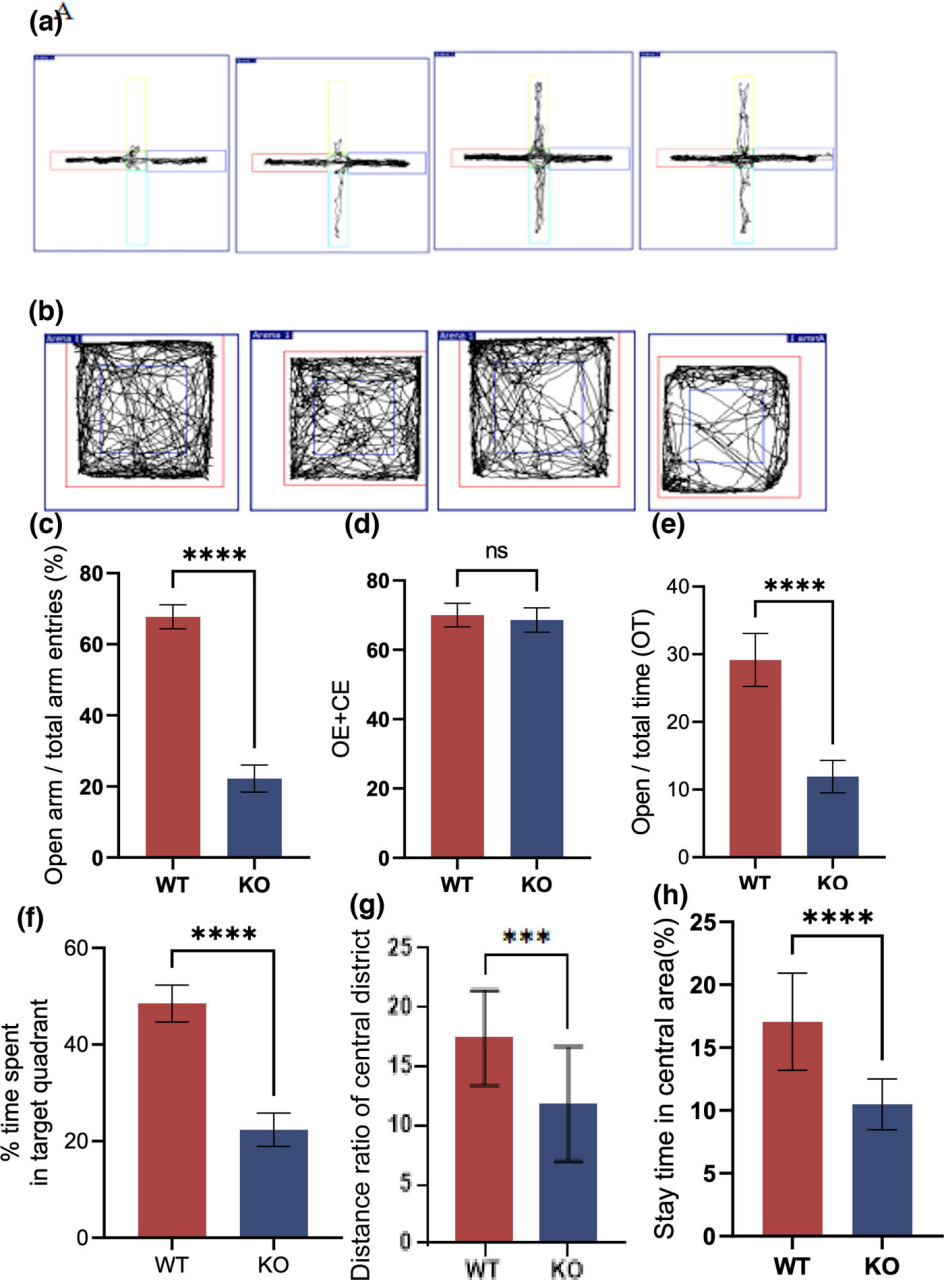
The results of elevated plus maze and open‐field experiments in mice showed that *MEX3C* knockout (KO) mice showed increased and decreased anxiety. (a) The activity route of the elevated plus maze experiment; (b) the activity route of the open‐field experiment; (c) the percentage of the times of entering the open arm of the elevated plus maze to the total number of arms entering OE (wild‐type [WT], *n* = 20, OE = 25.17% ± 10.3%; KO, *n* = 20, OE = 22.24% ± 3.81%, *p* = .0001); (D) the total number of times of entering the open and closed arms (WT, *n* = 20, times = 70.05 ± 3.41; KO, *n* = 20, times = 568.60 ± 3.49, *p* = .1915); (E) percentage of residence time OT in the open arm (WT, *n* = 20, OT = 29.16% ± 3.91%; KO, *n* = 20, OT = 11.90% ± 2.40%, *p* < .0001); (f) open‐field experiment into the central region time (WT, *n* = 20, time = 87.28 ± 23.95 s, KO, *n* = 20, time = 40.25 ± 22.07 s, *p* < .0001); (g) central area distance/total distance (WT, *n* = 20, percentage = 17.44% ± 4%; KO, *n* = 20, percentage = 11.85% ± 4.86%, *p* = .0003); (H) center zone residence time/total time (WT, *n* = 20, percentage = 17.00% ± 3.86%; KO, *n* = 20, percentage = 10.5% ± 2%, *p* < .0001). Data are mean ± standard deviation (SD). *^ns^p* > .05, ***p < .001, ****p < .0001.

After demonstrating that the loss of the *MEX3C* gene causes cognitive learning impairment in mice, we asked whether the loss of the gene affects other brain functions. To answer this question, we conducted an elevated plus maze experiment (Figure 4a) and a mice experiment (Figure 4b). In the elevated plus maze, there was no difference in motility between the mice in the WT group and those in the KO group (Figure 4d), but the depressive state of the mice in the KO group was significantly increased, that is, the activity time (OT) of the mice in the open arm was significantly increased in the KO group, and the number of times of entering the open arm (OE) was significantly lower than that of the mice in the WT group (Figure 4c,e). Also in the field experiment, there was no significant difference in the distance and speed of the mice in the *MEX3C*‐KO group, suggesting that the deletion of the *MEX3C* gene did not affect the exercise capacity of the mice. However, the *MEX3C*‐KO mice entered the central area 19.10 ± 9.48 times, stayed in the central area for 10.50 ± 2.0 s, and spent 40.25% ± 22.07% of their movement time in the central area, all of which were significantly lower compared with the results in the WT group. All these indicators were negatively correlated with anxiety‐like behavior, suggesting that the loss of the *MEX3C* gene can lead to increased anxiety‐like behavior (Figure 4g,h).

### 3.5 Deletion of the *MEX3C* gene changes Nissl bodies in neuroblasts

**FIGURE 5:**
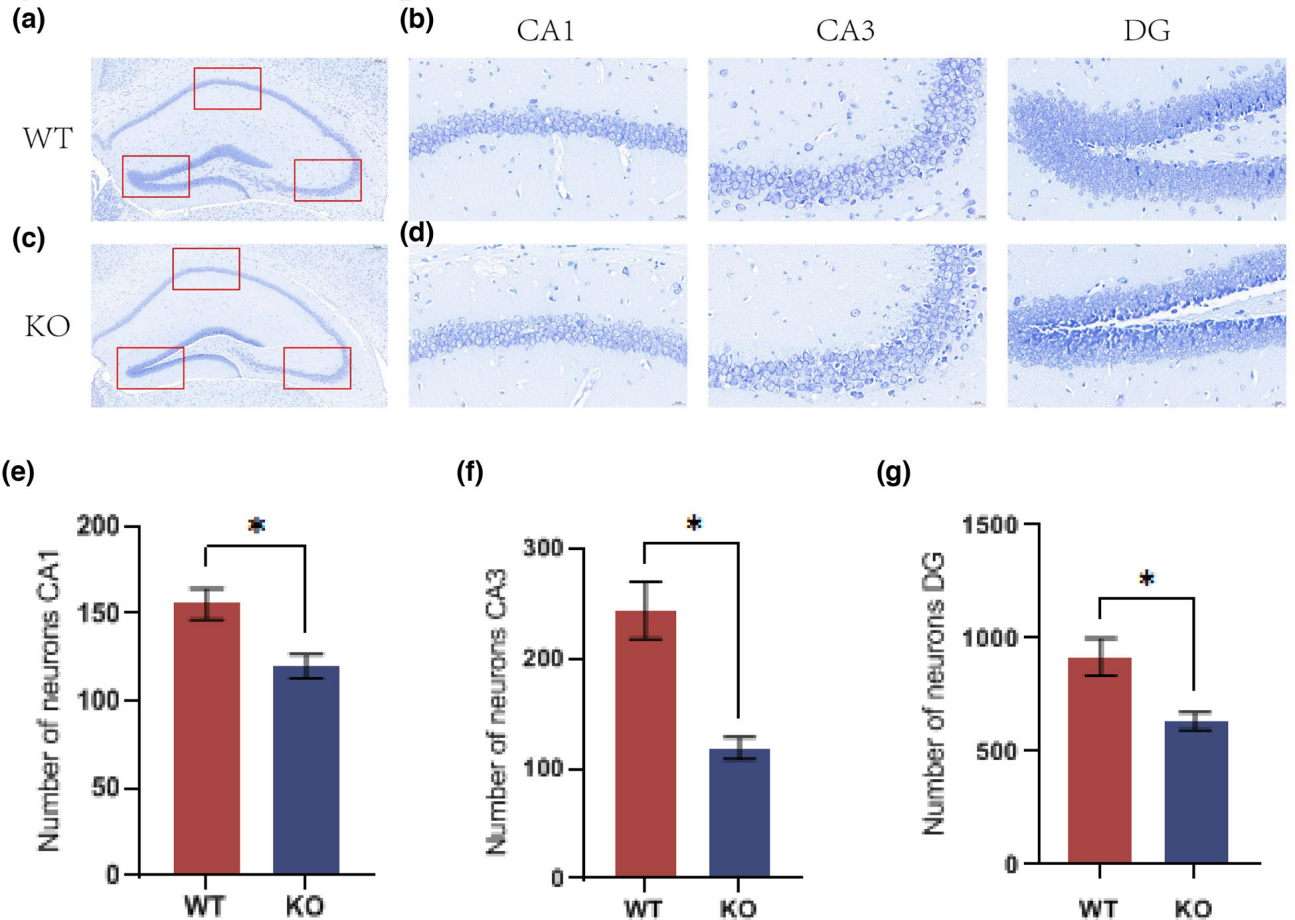
Nissl staining results showed that the number of neurons in the hippocampus of *MEX3C* knockout (KO) mice decreased. (a) Nissl staining results of hippocampus of mice in KO group (scale bar, 100 μm); (b) Nissl staining results of hippocampus in each area of KO group (scale bar, 20 μm); (c) Nissl staining results of hippocampus of mice in wild‐type (WT) group (scale bar, 100 μm); (d) Nissl staining results of each area of hippocampus in WT group (scale bar, 20 μm); (e) number of positive neurons in CA1 area (WT, *n* = 3, number = 155.6 ± 9.03; KO, *n* = 3, number = 119.8 ± 6.97, *p* = .0472); (f) number of positive neurons in CA3 area (WT, *n* = 3, number = 243.62 ± 25.91; KO, *n* = 3, number = 118.73 ± 9.97, *p* = .0238); (G) number of positive neurons in DG area (WT, *n* = 3, number = 917.7 ± 82.9; KO, *n* = 3, number = 633.67 ± 39.86, *p* = .0482). Data are mean ± standard deviation (SD). *p < .05.

Through the above behavioral experiments, we proved that the deletion of the *MEX3C* gene resulted in learning and memory impairment and increased anxiety‐like behaviors in mice. A Nissl body is a unique structure of neurons; its main function is to synthesize related proteins, such as the renewal of certain components in neuron cells and the production of neurotransmitters and enzymes, the latter of which is one of the indicators reflecting the degree of neuronal activity (Cheng et al., 2010). Nissl staining can effectively evaluate neuronal damage. As shown in Figure 5, the Nissl bodies in the CA1 and CA3 regions of the hippocampi of the mice in the WT group were dark blue, large in number, and arranged neatly. However, the Nissl bodies in the hippocampal CA1 and CA3 regions of the mice in the KO group were lighter in color, decreased in number, arranged disorderly, and were blurred or even absent.

### 3.6 Loss of the *MEX3C* gene reduces the number of mature neuron cells

**FIGURE 6:**
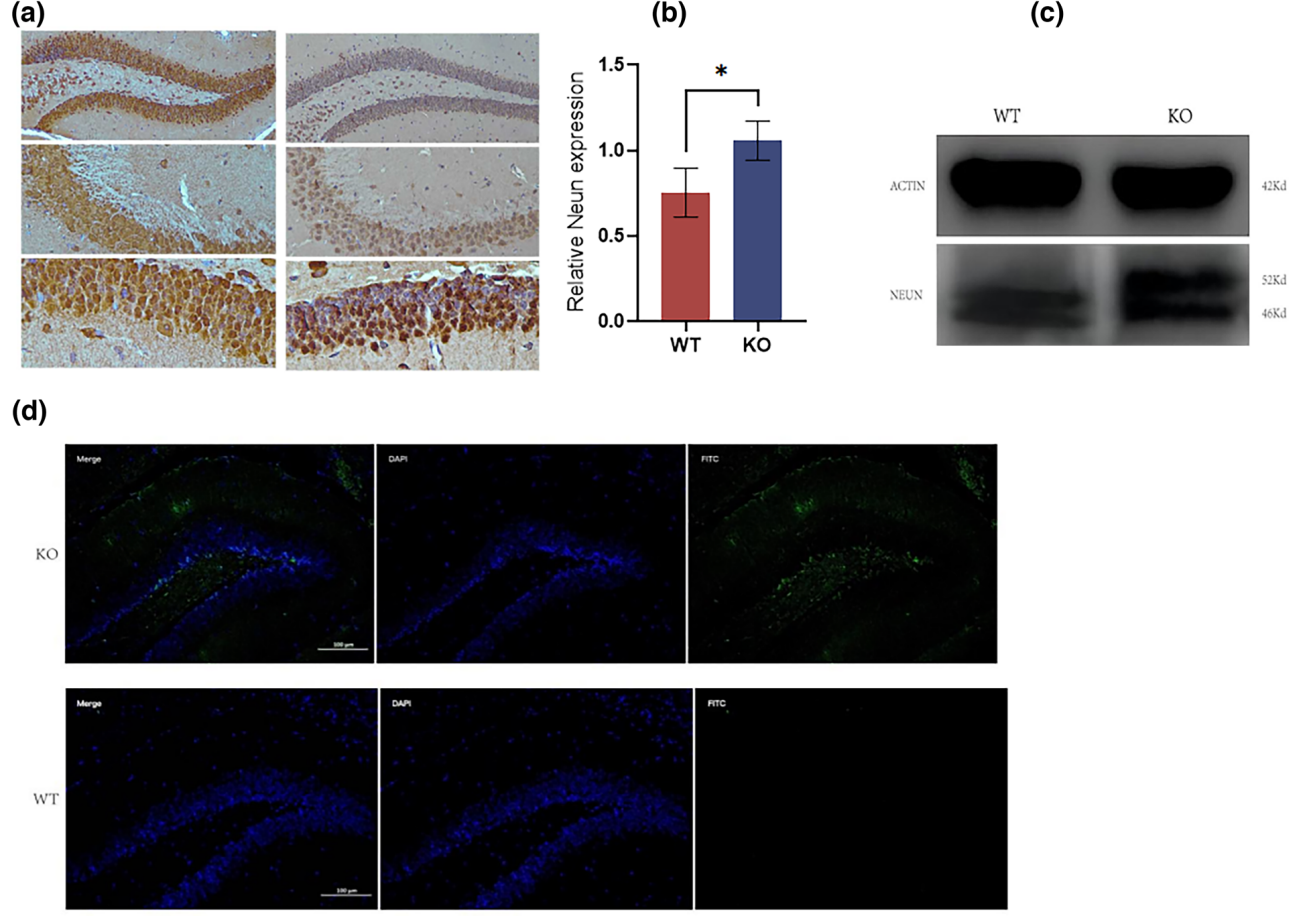
NEUN immunohistochemical staining and TUNNEL apoptosis detection. (a) NEUN immunohistochemical staining results of hippocampal DG, CA1, and CA3 regions in wild‐type (WT) group; NEUN immunohistochemical staining results of hippocampal DG, CA1, and CA3 regions in knockout (KO) group; (b) OD value of NEUN protein Western blot (WB) (WT, *n* = 3, OD = 1.06 ± 0.11; KO, *n* = 3, OD = 0.75 ± 0.14, *p* = .0460); (c) NEUN protein WB results; (d) TUNNEL staining results. Compared with WT group, the number of apoptotic cells in the hippocampus of KO group was significantly increased. Data are mean ± standard deviation (SD). *p < .05.

To study the effect of the *MEX3C* gene on the maturity of neurons, we selected mice on day 50 (essentially, the brain development of mice at this stage has been completed) for NEUN immunohistochemical staining of the brain and hippocampus  (Figure 6a,b). The results showed that compared with the control group, the number of NEUN‐positive neurons in the KO group was significantly less than that in the WT group. We also validated this at the protein level, with Western blot (WB) results showing that the expression of the NEUN protein was significantly lower in the KO group than in the WT group (Figure 6c). In the KO group, there was a large number of immature cells in the brain and hippocampus. To clarify the reason for the growth restriction of these cells, we performed a supplementary TUNEL apoptosis test in this region and found a large number of apoptotic cells in the cerebral cortex and hippocampus of the KO‐group mice (Figure 6d).

### 3.7 Deletion of the *MEX3C* gene reduces the synaptic plasticity of neurons

Dendritic spines are functional protrusions on the dendrites of neurons and the main sites of excitatory synapse transmission in the central nervous system. They receive external stimuli and transmit signals into the cell body; their size, density, and morphology affect the function of neuronal circuits (Ultanir et al., 2007). Their morphological function is affected by many factors and is constantly adjusting and changing. This change is consistent with alterations in the synaptic structure to adapt to the external environment. Therefore, the plasticity of dendritic spines is an important index by which to evaluate the function of central nervous system synapses, which is closely related to the learning and memory function of the brain.

We statistically analyzed the density of dendrites and dendritic spines of the cortical area and hippocampal vertebral neurons and found significant differences in the number of total dendrites and dendritic spine densities of the cortical area and hippocampal vertebral neurons of KO mice. The total number of dendrites in KO mice was significantly reduced compared with that of WT mice; the number of total dendrites in KO mice was 90, and the number of total dendrites in WT mice was 120. Among them were 63 first‐level dendrites, 17 second‐level dendrites, and 10 third‐level dendrites.

Then, we selected secondary dendrites with a length of 20 μm to count the number of dendritic spines on dendritic segments to reflect the density of dendritic spines. The higher the number, the greater the density of dendritic spines. The experiment found that the density of dendritic spines on the cortical area and the hippocampus of the mice in the KO group were significantly lower than that in the WT group. The number of dendritic spines per 20 μm length on the secondary dendrites of cortical vertebral neurons in the WT group was 110, and the number of dendritic spines per 20 μm length on the secondary dendrites of hippocampal vertebral neurons in the WT group was 95. The number of dendritic spines per 20 μm length on the secondary dendrites of cortical vertebral neurons in the KO group was 85, and the number of dendritic spines per 20 μm length on the secondary dendrites of hippocampal vertebral neurons in the WT group was 64.

To explore whether *MEX3C* gene KO changes the morphology of dendritic spines, we analyzed the types of dendritic spines and found that their distribution in *MEX3C*‐KO mice changed, and the number of thick spines decreased significantly. The number of fine spinous processes was significantly increased.

**FIGURE 7:**
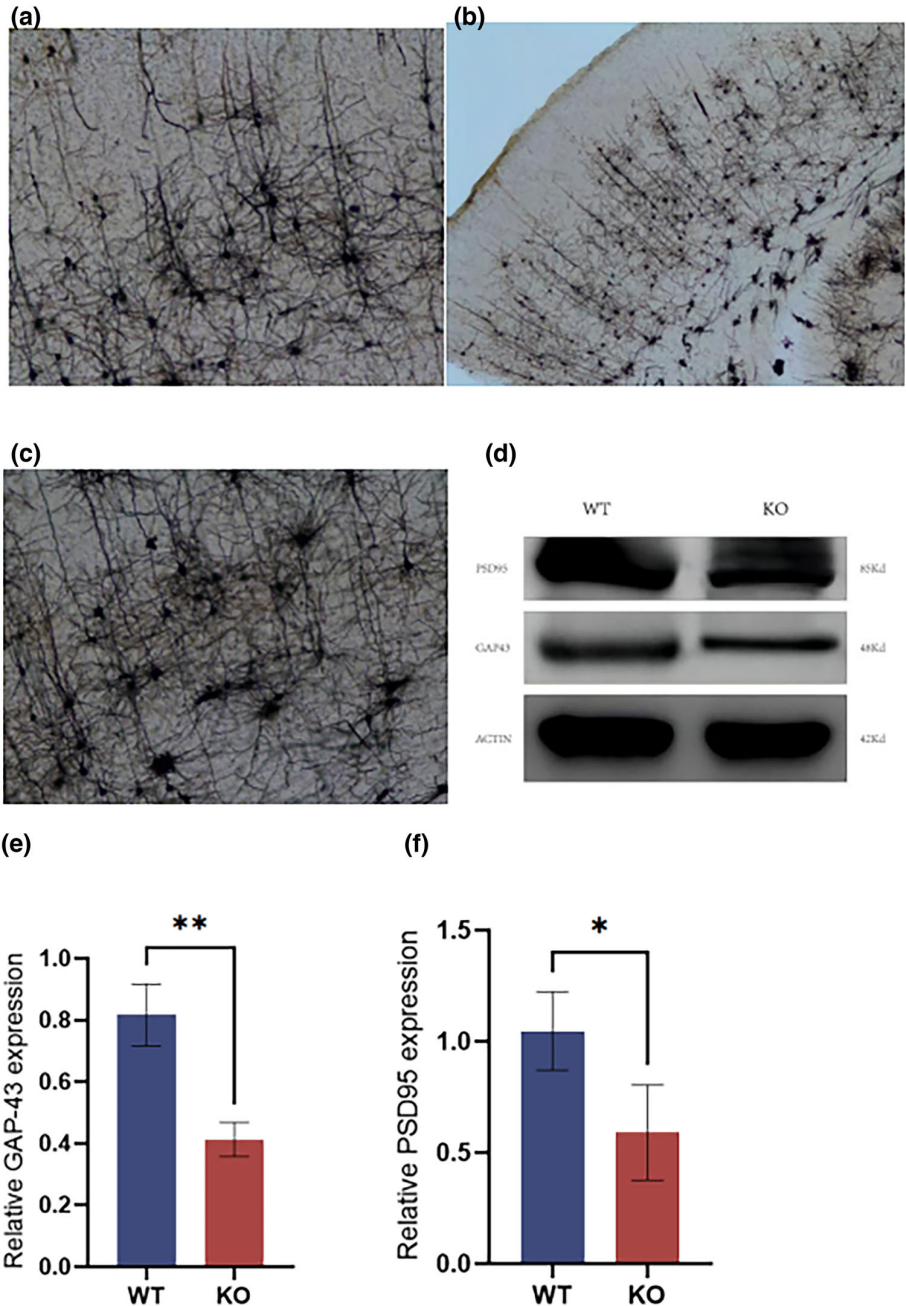
Results of total dendrites and Western blot (WB) results of postsynaptic density protein 95 (PSD‐95) and growth‐associated protein (GAP)‐43 proteins in cortical area and hippocampus of mice. (a) (b) (c) Results of total dendrites of vertebral neurons in cortical areas and hippocampus of mice; (d) WB results of PSD‐95 and GAP‐43 proteins (the ACTIN antibody panel in (d) is the same as that in Figure 6c, with the presentation split for organizational purposes.); (E) OD value of GAP‐43 protein WB experiments (wild‐type [WT], *n* = 3, OD = 0.82 ± 0.1; knockout [KO], *n* = 3, OD = 0.47 ± 0.41, *p* = .0036); (f) OD value of PSD‐95 protein WB experiment (WT, *n* = 3, OD = 1.05 ± 0.18; KO, *n* = 3, OD = 0.59 ± 0.21, *p* = .0461); data are mean ± standard deviation (SD). *p < .05, **p < .001.

Reduced levels of synapse‐associated proteins have been associated with memory impairment and abnormal synaptic plasticity (Giralt et al., 2012; Simmons et al., 2009). Therefore, we next examined the expression levels of several synapse‐associated proteins in the cerebral cortex and hippocampus, respectively. Interestingly, compared with the WT mice, the levels of synapse‐associated proteins, such as PSD‐95 and GAP‐43, were significantly lower in the KO mice. These findings suggest that memory deficits in *MEX3C*‐KO mice involve changes in the structure and plasticity of neuronal synapses (see Figure 7).

### 3.8 *MEX3C* gene deletion induces neuronal apoptosis and abnormal synaptic plasticity through autophagy inhibition

**FIGURE 8:**
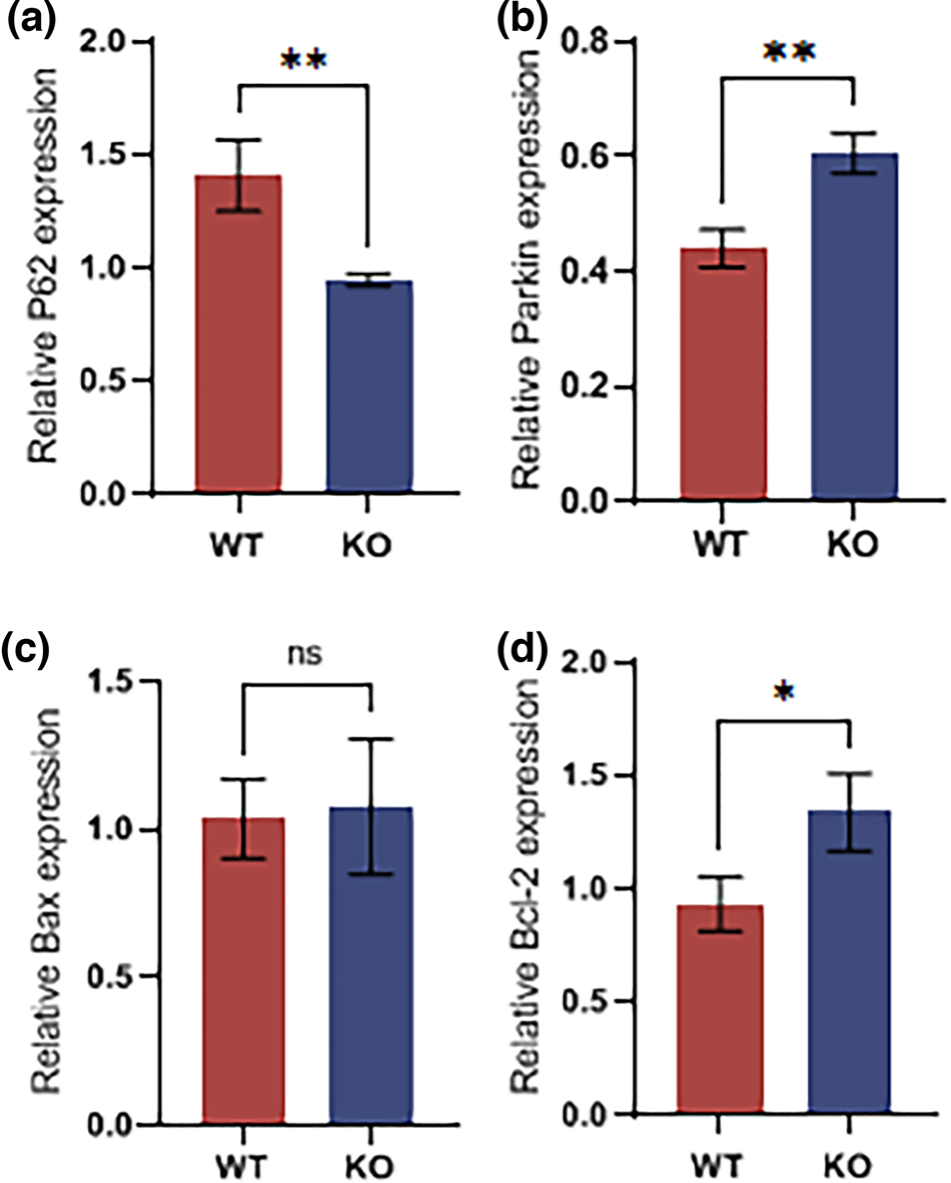
*MEX3C* knockout (KO) inhibits autophagy and abnormally activates apoptosis in hippocampal neurons, resulting in impaired cognitive function. (a) P62 protein expression in hippocampus (wild‐type [WT], *n* = 3, protein expression *n* = 1.41 ± 0.16; KO, *n* = 3, protein expression *n* = 0.94 ± 0.03, *p* = .0077); (b) Parkin protein expression (WT, *n* = 3, protein expression *n* = 1.41 ± 0.16; KO, *n* = 3, protein expression *n* = 0.94 ± 0.03, *p* = .0077); (c) Bax protein expression (WT, *n* = 3, protein expression *n* = 1.03 ± 0.13; KO, *n* = 3, protein expression *n* = 1.08 ± 0.23, *p* = .7987); (d) Bcl‐2 protein expression (WT, *n* = 3, protein expression *n* = 0.93 ± 0.12; KO, *n* = 3, protein expression level = 1.34 ± 0.17, *p* = .0281) data are mean ± standard deviation (SD). ^ns^ *p* > .05, *p < .05, **p < .01.

Our previous study found that the genetic deletion of the *MEX3C* gene caused the formation of neural tube defects in mouse embryos via the regulation of autophagy. Therefore, is cognitive impairment in mice caused by the deletion of the *MEX3C* gene also caused by the inhibition of autophagy resulting from the deletion of the *MEX3C* gene? To explore this question, we first examined the expression levels of several autophagy‐related proteins and found that the expression levels of P62 and Parkin proteins in the brains and hippocampi of KO mice were significantly lower than in those of WT mice. We analyzed the gray‐scale results of WB for both proteins (Figure 8a,b).

Autophagy and apoptosis control organelle and protein turnover, respectively. Autophagy normally blocks the induction of apoptosis, whereas apoptosis‐related caspase activation shuts down the autophagic process, that is, the relationship between autophagy and apoptosis is mutually inhibitory in the vast majority of cases. However, under certain pathological conditions, the relationship between autophagy and apoptosis may shift to one of reciprocal promotion (Mariño et al., 2014). For this reason, we detected the protein expression levels of Bax and Bcl‐2. The gray density WB results showed that the ratio of Bax/Bcl‐2 in the neuronal cells of the mice in the KO group was significantly higher than that in the WT group (Figure 8c,d); this was consistent with previous TUNEL staining results, indicating that *MEX3C* gene deletion enhanced neuronal apoptosis through autophagy inhibition, which, in turn, led to the formation of cognitive impairment in mice.

## 4 DISCUSSION

In this study, we explored the expression of the *MEX3C* gene in the mouse brain and demonstrated its role in cognitive function and emotional performance through behavioral and morphological experiments. We also investigated whether *MEX3C* loss mediates cognitive impairment and mood dysregulation by affecting autophagy and apoptosis in the brain. Our results showed that the *MEX3C* gene was expressed in the hippocampus of mice and was positively correlated with the differentiation and maturation of mature neurons and the maintenance of synaptic function. In the hippocampus of *MEX3C*‐null mice, on the one hand, NeuN and KI67 protein levels were decreased, as were neuronal proliferation and differentiation. On the other hand, the expression of synaptic plasticity‐related proteins, such as PSD‐95 and GAP‐43, also decreased, as did the plasticity of neuronal synapses. Finally, our data showed that P62 and Parkin autophagy‐related proteins decreased, whereas Bax and Bcl‐2 proteins increased. These results suggest that *MEX3C* deletion affects neuronal function and synaptic plasticity by inhibiting autophagy in hippocampal neurons, ultimately leading to impaired cognitive function and increased anxiety predisposition in the hippocampus.

The brain is the main part of the central nervous system and the most important part of the differentiation and development of the central nervous system during the embryonic period. The formation of its functions is subject to complex but fine regulation, and the regulation of genes is the most important regulatory factor (Ha et al., 2015; Schubert et al., 2015). Mammals not only have highly differentiated neural circuits to extract and process complex information but, more importantly, they also have high‐order social cognitive behaviors, such as learning and emotion regulation. Additionally, these higher behaviors are inseparable from the memory function of the hippocampus.

The *MEX3C* gene has been confirmed to be closely related to growth and development, especially in the central nervous system. Recent studies found that the *MEX3C* gene is involved in regulating the expression of PAX3 and Nestin proteins in the development of the embryonic neural tube in mice, thereby inhibiting the development and maturation of nerve cells in the neural tube and promoting apoptosis within (Du et al., 2020). In this study, we found that *MEX3C*‐KO mice had significantly increased learning time and decreased memory ability in the water maze, novel object recognition, and shuttle box experiments; although the mice in the KO group formed memory, their memory reproduction ability was significantly decreased, while in the elevated plus maze and open‐field experiments, the KO mice also showed increased anxiety. These results indicate that the deletion of the *MEX3C* gene causes the impairment of cognitive function and emotion regulation.

Interestingly, there was no significant difference in the weight of the two groups of mice, and there was no significant difference in the shape and weight of the brains of the two groups at different developmental stages. Therefore, the KO of the gene did not cause significant changes in the individual characteristics of the mice. This may be due to the existence of certain mechanisms that counteract the effects of *MEX3C* KO on mouse characterization, such as a significant increase in glial cells.

Mature neurons in the brain are the most basic unit of signal reception and transmission. The interconnection and communication between neurons constitute the complex and precise neural network structure of the brain (Sakurai & Katz, 2009). Studies have shown that abnormal neuronal and synaptic plasticity is one of the underlying mechanisms of cognitive function and anxiety behavior (Dani & Broadie, 2012). Therefore, we next studied whether the deletion of the *MEX3C* gene causes abnormal reductions in the number, function, synaptic density, and plasticity of mouse neurons.

The number of mature neurons is crucial to the cognitive function of memory. Through brain research on *MEX3C*‐KO mice, we found that the number of neurons in the hippocampus and cerebral cortex of *MEX3C* gene‐deficient mice decreased; their arrangement was chaotic, their maturity was decreased, and the number of Nissl bodies was also decreased. The extraction of external information, processing, and transmission depend on the synaptic plasticity of neuronal dendrites. Although there is no report on *MEX3C* and synaptic plasticity at this stage, several studies have shown that E3 ubiquitin ligase regulates synaptic plasticity by interfering with various ubiquitination processes (Gao et al., 2017; Ma et al., 2022). MEX‐3 RNA‐binding family member C (*MEX3C*), the MEX‐3 RNA‐binding protein family, has two tandem KH RNA‐binding domains at its N terminus and a RING domain with E3 ubiquitin ligase activity at its C terminus, which is involved in much important posttranscriptional regulation. These studies suggest that *MEX3C* must be related to synaptic plasticity and synapse‐associated proteins. The results of WB showed that the expression of synapse‐associated proteins PSD‐95 and GAP‐43 decreased. PSD‐95 can modulate synaptic strength and aspects of activity‐dependent plasticity (El‐Husseini et al., 2002), whereas GAP‐43 is a protein kinase C‐activated phosphorylated protein often associated with axonal plasticity and regeneration (Hung et al., 2016).

Our research group's previous work showed that the KO of the *MEX3C* gene affects the autophagy function of cells and mitochondria, and autophagy is the main degradation system in the cells of organisms. Through this life activity, some substances in cells, such as misfolded proteins and dysfunctional organelles, are delivered and degraded in lysosomes to maintain cell renewal and homeostasis (Mizushima & Komatsu, 2011). Similar to cell differentiation and death, autophagy is a key link in the pathogenesis of many diseases, and the abnormal activation or inhibition of autophagy is its pathogenic mechanism. It has been reported that autophagy inhibition may play a crucial role in cognitive dysfunction in neurological diseases (Pozniak et al., 2000). It is worth noting that mice with *MEX3C* gene deletion have obvious autophagy inhibition and increased apoptosis. According to the results of WB, we found that p62 and Parkin proteins decreased in the KO group, although Bax and Bcl‐2 proteins increased.

Cell apoptosis refers to genetically programmed cell death, which plays an important role in the growth and development of organisms (Ha et al., 2015; Mariño et al., 2014). However, the abnormal activation of apoptosis may have negative consequences, especially in some nonrenewable cells, such as neurons. Current studies suggest that apoptosis may occur and be strongly associated with the onset and progression of neurodegenerative diseases (Cusack et al., 2013; Saito et al., 2005). In the present study, the deletion of the *MEX3C* gene led to a significant increase in the number of hippocampal TUNEL‐positive cells.

A major apoptosis pathway is the mitochondrial pathway (Gressner et al., 2005). The Bcl‐2 protein is mainly responsible for inhibiting apoptosis, whereas the Bax protein is responsible for promoting it. The experimental results showed that the KO of the *MEX3C* gene had little effect on the expression of the Bcl‐2 protein, although it significantly promoted the expression of the Bax protein. These results suggest that the mitochondrial pathway of apoptosis may be involved in *MEX3C*‐KO‐activated neuronal apoptosis. The normal physiology of organisms depends on the normal physiology of cells. *MEX3C* KO causes the abnormal activation of neuronal apoptosis, resulting in increased or premature neuronal apoptosis, which, in turn, causes the impairment of hippocampal function and cognition.

The results of this study show that *MEX3C* gene KO reduced synaptic plasticity in various regions of the hippocampus, thus affecting the function of the hippocampus and eventually leading to a decline in cognitive function. Compared with WT mice, *MEX3C*‐KO mice showed an increase in anxiety‐like behavior during minefield and maze tests. The results provide a basis for further research to clarify the induction by *MEX3C* of cognitive dysfunction in mice by inhibiting autophagy. However, the specific mechanism needs further exploration. The molecular mechanism related to autophagy or apoptosis caused by *MEX3C* KO and the involved biochemical processes (such as ubiquitination and phosphorylation) will be the focus of our research group's next investigation.

## 5 CONCLUSION

In conclusion, our experimental results are consistent with the expected hypothesis that *MEX3C* KO may lead to a decrease in the number and maturity of neurons, and the decreased expression of SD95 and GAP‐43 reduces the plasticity and complexity of hippocampal synapses, which together lead to cognitive decline and increased anxiety in mice. The specific mechanism of *MEX3C* deficiency leading to mental retardation in offspring may be related to the inhibition of neuronal autophagy and the abnormal activation of apoptosis. The effect of *MEX3C* KO on the plasticity of the nervous system was reported herein for the first time, and we have a new understanding of the mechanism of *MEX3C* on the development of the nervous system. In the future, we will use ChIP‐seq technology to explore deeper mechanisms and provide corresponding theoretical support for clinical work.

### PEER REVIEW

The peer review history for this article is available at https://publons.com/publon/10.1002/brb3.3245.

## Data Availability

All data generated or analyzed during this study are included in this article.
